# A triclinic polymorph of poly[[bis­[μ-1,2-bis­(pyridin-4-yl)ethene-κ^2^ *N*:*N*′]bis­(thio­cyanato-κ*N*)cobalt(II)] 1,2-bis­(pyridin-4-yl)ethene monosolvate]

**DOI:** 10.1107/S1600536812019009

**Published:** 2012-05-05

**Authors:** Susanne Wöhlert, Inke Jess, Christian Näther

**Affiliations:** a Institut für Anorganische Chemie, Christian-Albrechts-Universität Kiel, Max-Eyth-Strasse 2, 24118 Kiel, Germany

## Abstract

In the crystal structure of the title compound, [Co(NCS)_2_(C_12_H_10_N_2_)_2_]·C_12_H_10_N_2_, the Co^II^ cations are octa­hedrally coordinated by two terminally *N*-bonded thio­cyanate anions and four 1,2-bis­(pyridin-4-yl)ethene (bpe) ligands. The asymmetric unit consists of three crystallographically independent Co^II^ cations, six thio­cyanate anions and six coordinating bpe ligands in general positions. Additionally, three non-coordin­ating bpe ligands are present in the asymmetric unit with two of them located on a center of inversion. The Co^II^ cations are connected by the bpe ligands into layers parallel to the *bc* plane. The crystal investigated was non-merohedrically twinned, with a fractional contribution of 0.261 (2) for the minor domain.

## Related literature

For background to this work see: Boeckmann & Näther (2010 ▶), Wriedt *et al.* (2009 ▶). For the monoclinic polymorph of the title compound, see: Wöhlert *et al.* (2011 ▶).
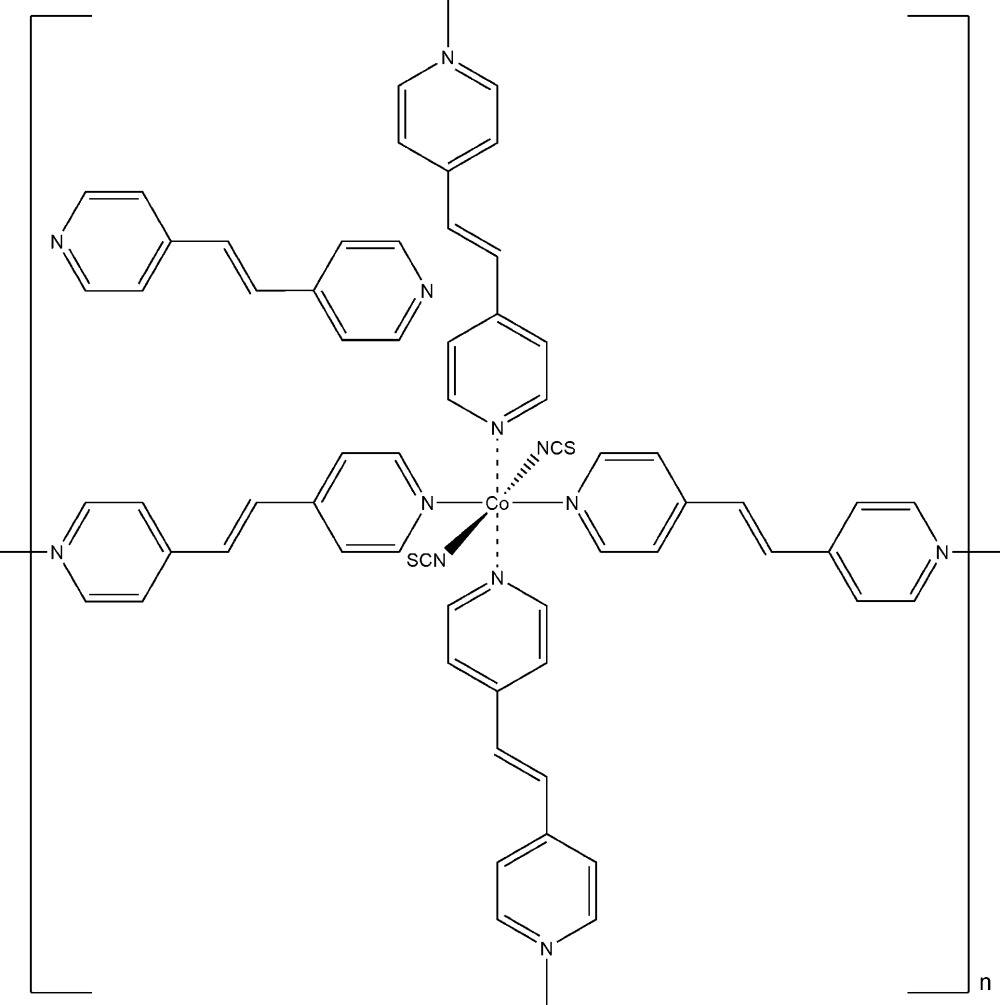


## Experimental

### 

#### Crystal data

[Co(NCS)_2_(C_12_H_10_N_2_)_2_]·C_12_H_10_N_2_
*M* *_r_* = 721.75
Triclinic, 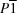

*a* = 13.7954 (14) Å
*b* = 13.8916 (13) Å
*c* = 27.848 (2) Å
α = 99.892 (10)°
β = 90.547 (11)°
γ = 91.269 (11)°
*V* = 5255.7 (8) Å^3^
*Z* = 6
Mo *K*α radiation
μ = 0.65 mm^−1^
*T* = 170 K
0.3 × 0.2 × 0.2 mm

#### Data collection

Stoe IPDS-1 diffractometer
15899 measured reflections
15899 independent reflections
11067 reflections with *I* > 2σ(*I*)
θ_max_ = 24.1°

#### Refinement

*R*[*F* ^2^ > 2σ(*F* ^2^)] = 0.054
*wR*(*F* ^2^) = 0.166
*S* = 1.03
15899 reflections
1326 parameters
H-atom parameters constrained
Δρ_max_ = 0.84 e Å^−3^
Δρ_min_ = −0.68 e Å^−3^

### 

Data collection: *X-AREA* (Stoe & Cie, 2008 ▶); cell refinement: *X-AREA*; data reduction: *X-RED32* (Stoe & Cie, 2008 ▶); program(s) used to solve structure: *SHELXS97* (Sheldrick, 2008 ▶); program(s) used to refine structure: *SHELXL97* (Sheldrick, 2008 ▶); molecular graphics: *XP* (Sheldrick, 2008 ▶) and *DIAMOND* (Brandenburg, 2011 ▶); software used to prepare material for publication: *CIFTAB* (Sheldrick, 2008 ▶).

## Supplementary Material

Crystal structure: contains datablock(s) I, global. DOI: 10.1107/S1600536812019009/bt5886sup1.cif

Structure factors: contains datablock(s) I. DOI: 10.1107/S1600536812019009/bt5886Isup2.hkl

Additional supplementary materials:  crystallographic information; 3D view; checkCIF report

## Figures and Tables

**Fig. 1.**
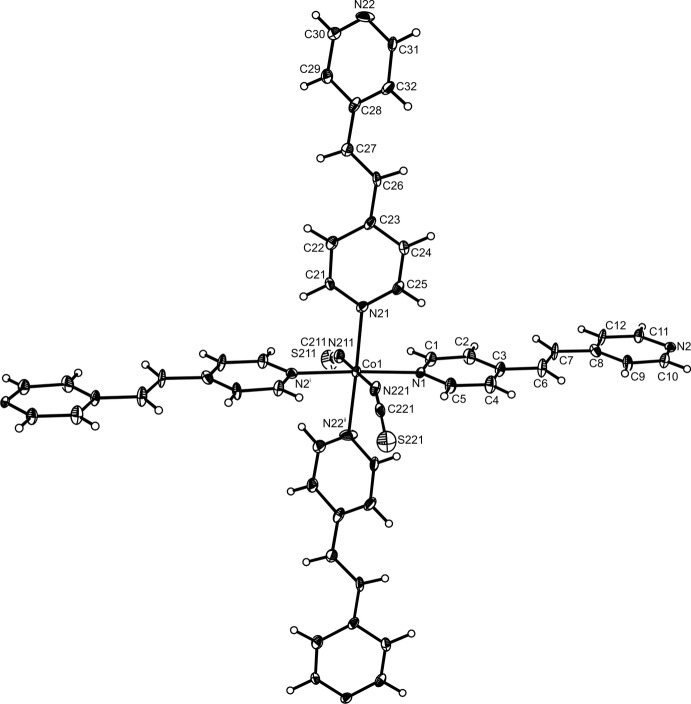
: Environment of Co1 with labelling and displacement ellipsoids drawn at the 50 % probability level. Symmetry codes: i = x, y-1, z; ii = x+1, y, z. For clarity the non-coordinated bpe ligands are omitted.

**Fig. 2.**
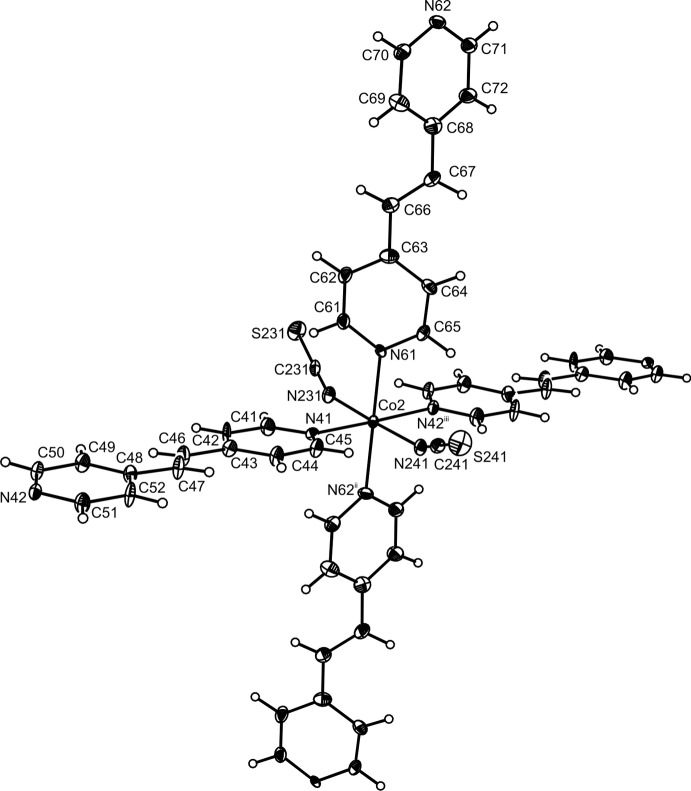
: Environment of Co2 with labelling and displacement ellipsoids drawn at the 50 % probability level. Symmetry codes: ii = x+1, y, z; iii = x, y+1, z. For clarity the non-coordinated bpe ligands are omitted.

**Fig. 3.**
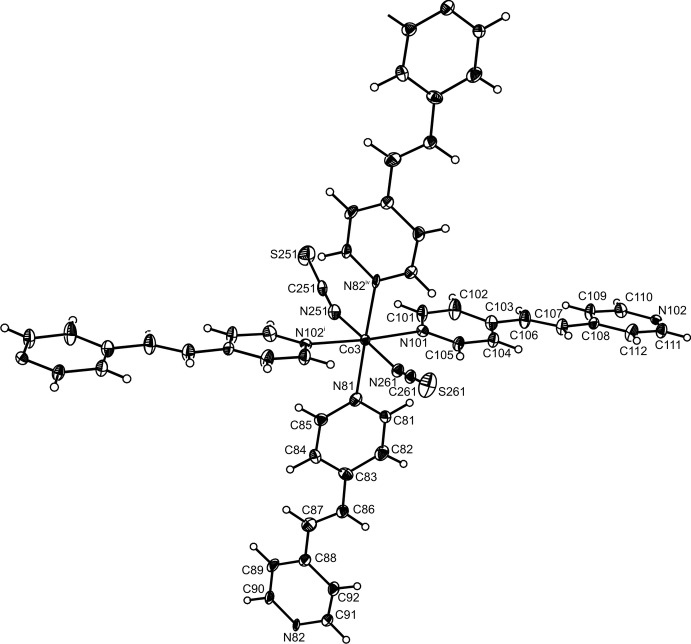
: Environment of Co3 with labelling and displacement ellipsoids drawn at the 50 % probability level. Symmetry codes: i = x, y-1, z; iv = x-1, y, z. For clarity the non-coordinated bpe ligands are omitted.

**Fig. 4.**
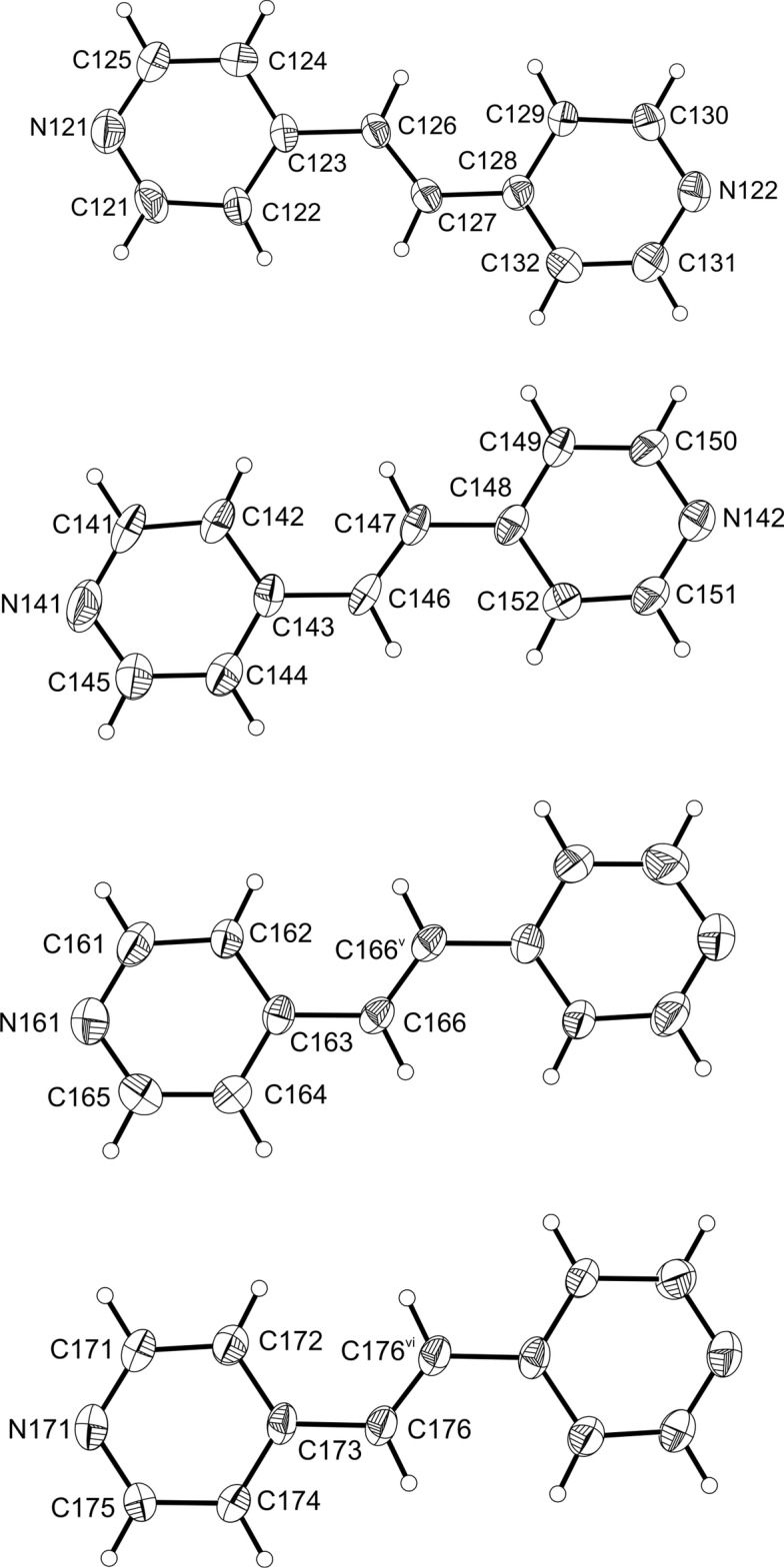
: View of the non-coordinated 1,2-bis(pyridine-4-yl)ethyene ligands with labelling and displacement ellipsoids drawn at the 50 % probability level. Symmetry codes: v = -x+1, -y+2, -z+1; vi = -x+1, -y, -z.

**Fig. 5.**
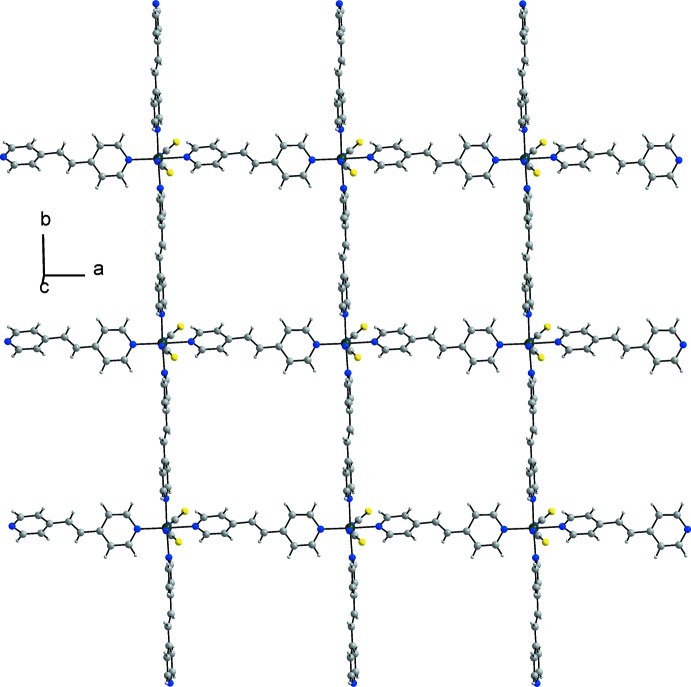
: Crystal structure of the title compound with view approximately along the crystallographic c-axis. The non-coordinated bpe ligands are omitted for clarity.

## supplementary crystallographic information

### Comment

Recently we have reported on our investigations on the synthesis, structures and properties of new coordination polymers based on paramagnetic transition metal and *N*-donor co-ligands, in which the transition metal anions are linked into chains by thiocyanato anions (Wriedt *et al.*, 2009 and Boeckmann & Näther, 2010 as well as Wöhlert *et al.*, 2011). In the course of these investigations we have reported on a cobalt-thiocyanato compound with bpe (1,2-bis(pyridine-4-yl)ethyene) as ligand that shows a slow relaxation of the magnetization (Wöhlert *et al.*, 2011). This compound can be prepared by thermal decomposition of the precursor [bis(thiocyanato-*N*)-bis(1,2-bis(pyridine-4-yl)ethyene-*N*)-cobalt(II)]-1,2-bis(pyridine-4-yl)ethyene-solvate. In further investigations we have obtained a second polymorphic modification of this precursor which was characterized by single crystal X-ray diffraction.

In the crystal structure of the title compound, each of the three cobalt(II) cation is coordinated by two terminally *N*-bonded thiocyanato anions and four bpe ligands (Fig. 1–4). The CoN_6_ octahedrons are slightly distorted with distances ranging from 2.051 (5) Å to 2.245 (3). The angles arround the metal atoms are in the range of 87.39 ° to 178.91 (15) °. The cobalt(II) cations are linked by the bpe ligands into chains that are further connected into layers by additional coligands (Fig. 5). The layers are stacked in order that channels are formed, in which non-coordinated bpe molecules are located. The Co—Co distances within the chains are 13.7954 (16) Å and 13.8916 (15) Å.

### Experimental

Cobalt thiocyanate (Co(NCS)_2_) and 1,2-bis(pyridine-4-yl)ethyene (bpe) were obtained from Alfa Aesar, Sigma Aldrich respectively. All chemicals were used without further purification. 0.15 mmol (28.9 mg) Co(NCS)_2_ and 0.6 mmol (108.4 mg) bpe were reacted with 1 ml H_2_O in a closed test-tube at 120 ° C for three days. On cooling orange block-shaped single crystals of the title compound were obtained.

### Refinement

H atoms were positioned with idealized geometry and were refined isotropically with *U*_iso_(H) = 1.2*U*_eq_(C) and C—H distances of 0.95 Å using a riding model. The crystal investigated was non-merohedrically twinned. The twin matrix was calculated from the orientation matrices of both individuals and afterwards a new HKL file in HKLF-5 format was generated and the structure was refined using the HKLF-5 option in SHELXL-97 [BASF parameter: 0.261 (2)].

### Crystal data

**Table:**

[Co(NCS)_2_(C_12_H_10_N_2_)_2_]·C_12_H_10_N_2_	*Z* = 6
*M**_r_* = 721.75	*F*(000) = 2238
Triclinic, *P*1	*D*_x_ = 1.368 Mg m^−^^3^
Hall symbol: -P 1	Mo *K*α radiation, λ = 0.71073 Å
*a* = 13.7954 (14) Å	Cell parameters from 15899 reflections
*b* = 13.8916 (13) Å	θ = 1.6–24.1°
*c* = 27.848 (2) Å	µ = 0.65 mm^−^^1^
α = 99.892 (10)°	*T* = 170 K
β = 90.547 (11)°	Block, orange
γ = 91.269 (11)°	0.3 × 0.2 × 0.2 mm
*V* = 5255.7 (8) Å^3^	

### Data collection

**Table:**

Stoe IPDS-1 diffractometer	11067 reflections with *I* > 2σ(*I*)
Radiation source: fine-focus sealed tube	*R*_int_ = 0.000
Graphite monochromator	θ_max_ = 24.1°, θ_min_ = 1.6°
phi scans	*h* = −15→15
15899 measured reflections	*k* = −15→15
15899 independent reflections	*l* = −30→30

### Refinement

**Table:**

Refinement on *F*^2^	Secondary atom site location: difference Fourier map
Least-squares matrix: full	Hydrogen site location: inferred from neighbouring sites
*R*[*F*^2^ > 2σ(*F*^2^)] = 0.054	H-atom parameters constrained
*wR*(*F*^2^) = 0.166	*w* = 1/[σ^2^(*F*_o_^2^) + (0.1032*P*)^2^] where *P* = (*F*_o_^2^ + 2*F*_c_^2^)/3
*S* = 1.03	(Δ/σ)_max_ = 0.001
15899 reflections	Δρ_max_ = 0.84 e Å^−^^3^
1326 parameters	Δρ_min_ = −0.68 e Å^−^^3^
0 restraints	Extinction correction: *SHELXL97* (Sheldrick, 2008), Fc^*^=kFc[1+0.001xFc^2^λ^3^/sin(2θ)]^-1/4^
Primary atom site location: structure-invariant direct methods	Extinction coefficient: 0.0054 (5)

### Special details

**Table:**

Geometry. All esds (except the esd in the dihedral angle between two l.s. planes) are estimated using the full covariance matrix. The cell esds are taken into account individually in the estimation of esds in distances, angles and torsion angles; correlations between esds in cell parameters are only used when they are defined by crystal symmetry. An approximate (isotropic) treatment of cell esds is used for estimating esds involving l.s. planes.
Refinement. Refinement of F^2^ against ALL reflections. The weighted R-factor wR and goodness of fit S are based on F^2^, conventional R-factors R are based on F, with F set to zero for negative F^2^. The threshold expression of F^2^ > 2sigma(F^2^) is used only for calculating R-factors(gt) etc. and is not relevant to the choice of reflections for refinement. R-factors based on F^2^ are statistically about twice as large as those based on F, and R- factors based on ALL data will be even larger.

### Fractional atomic coordinates and isotropic or equivalent isotropic displacement parameters (Å^2^)

**Table:**

	*x*	*y*	*z*	*U*_iso_*/*U*_eq_	
Co1	0.10026 (4)	0.52321 (4)	0.24971 (2)	0.01071 (17)	
N1	0.1118 (3)	0.6860 (3)	0.25417 (14)	0.0120 (8)	
C1	0.0997 (3)	0.7288 (3)	0.21527 (19)	0.0179 (11)	
H1	0.0913	0.6880	0.1844	0.021*	
C2	0.0988 (4)	0.8297 (3)	0.21715 (19)	0.0207 (11)	
H2	0.0879	0.8562	0.1884	0.025*	
C3	0.1138 (3)	0.8907 (3)	0.26146 (18)	0.0155 (10)	
C4	0.1286 (4)	0.8463 (3)	0.30218 (19)	0.0218 (11)	
H4	0.1401	0.8854	0.3333	0.026*	
C5	0.1265 (3)	0.7457 (3)	0.29740 (18)	0.0179 (11)	
H5	0.1359	0.7173	0.3257	0.021*	
C6	0.1144 (4)	0.9989 (3)	0.2683 (2)	0.0236 (12)	
H6	0.1330	1.0338	0.2995	0.028*	
C7	0.0915 (4)	1.0499 (3)	0.23507 (19)	0.0207 (11)	
H7	0.0711	1.0145	0.2042	0.025*	
C8	0.0941 (3)	1.1579 (3)	0.24068 (19)	0.0173 (11)	
C9	0.1088 (4)	1.2200 (3)	0.28521 (19)	0.0196 (11)	
H9	0.1190	1.1942	0.3143	0.024*	
C10	0.1083 (3)	1.3204 (3)	0.28622 (18)	0.0179 (11)	
H10	0.1178	1.3619	0.3168	0.022*	
C11	0.0818 (3)	1.3017 (3)	0.20417 (18)	0.0174 (11)	
H11	0.0723	1.3292	0.1755	0.021*	
C12	0.0809 (4)	1.2006 (3)	0.19969 (19)	0.0199 (11)	
H12	0.0714	1.1608	0.1686	0.024*	
N2	0.0951 (3)	1.3627 (3)	0.24665 (14)	0.0132 (8)	
N21	−0.0598 (3)	0.5304 (3)	0.25350 (15)	0.0145 (9)	
C21	−0.1180 (3)	0.4489 (3)	0.24838 (18)	0.0161 (10)	
H21	−0.0879	0.3874	0.2411	0.019*	
C22	−0.2179 (3)	0.4489 (4)	0.2530 (2)	0.0214 (12)	
H22	−0.2540	0.3890	0.2497	0.026*	
C23	−0.2647 (3)	0.5377 (3)	0.26261 (19)	0.0159 (11)	
C24	−0.2043 (3)	0.6227 (3)	0.26913 (19)	0.0197 (11)	
H24	−0.2320	0.6854	0.2772	0.024*	
C25	−0.1057 (3)	0.6149 (3)	0.26386 (17)	0.0162 (10)	
H25	−0.0677	0.6736	0.2679	0.019*	
C26	−0.3699 (4)	0.5482 (3)	0.26806 (19)	0.0206 (11)	
H26	−0.3934	0.6047	0.2883	0.025*	
C27	−0.4348 (3)	0.4805 (4)	0.2454 (2)	0.0202 (11)	
H27	−0.4113	0.4219	0.2269	0.024*	
C28	−0.5394 (4)	0.4938 (3)	0.24813 (19)	0.0183 (11)	
C29	−0.5996 (4)	0.4351 (4)	0.2128 (2)	0.0217 (11)	
H29	−0.5725	0.3874	0.1884	0.026*	
C30	−0.6986 (3)	0.4484 (4)	0.2146 (2)	0.0220 (12)	
H30	−0.7381	0.4097	0.1902	0.026*	
C31	−0.6837 (3)	0.5666 (3)	0.28201 (19)	0.0181 (11)	
H31	−0.7133	0.6126	0.3063	0.022*	
C32	−0.5844 (3)	0.5598 (3)	0.2831 (2)	0.0211 (11)	
H32	−0.5470	0.6003	0.3078	0.025*	
N22	−0.7414 (3)	0.5108 (3)	0.24776 (17)	0.0262 (11)	
S211	0.15370 (12)	0.51234 (11)	0.07889 (6)	0.0344 (4)	
C211	0.1200 (3)	0.5073 (3)	0.1342 (2)	0.0183 (11)	
N211	0.0969 (3)	0.5049 (3)	0.17461 (16)	0.0176 (9)	
S221	0.18076 (12)	0.54030 (11)	0.41796 (5)	0.0343 (4)	
C221	0.1363 (3)	0.5428 (3)	0.36419 (19)	0.0152 (10)	
N221	0.1052 (3)	0.5440 (3)	0.32487 (16)	0.0160 (9)	
Co2	0.90452 (4)	0.17932 (4)	0.41476 (2)	0.01004 (17)	
N41	0.9056 (3)	0.0185 (3)	0.41222 (14)	0.0125 (8)	
C41	0.9135 (4)	−0.0417 (3)	0.36949 (19)	0.0196 (11)	
H41	0.9188	−0.0137	0.3408	0.024*	
C42	0.9142 (4)	−0.1420 (3)	0.36485 (19)	0.0220 (12)	
H42	0.9204	−0.1811	0.3336	0.026*	
C43	0.9059 (3)	−0.1863 (3)	0.40576 (18)	0.0155 (10)	
C44	0.8979 (4)	−0.1246 (3)	0.45000 (19)	0.0191 (11)	
H44	0.8931	−0.1509	0.4792	0.023*	
C45	0.8968 (4)	−0.0246 (3)	0.45181 (19)	0.0191 (11)	
H45	0.8894	0.0159	0.4826	0.023*	
C46	0.9080 (4)	−0.2937 (3)	0.3996 (2)	0.0218 (11)	
H46	0.9206	−0.3278	0.3678	0.026*	
C47	0.8941 (4)	−0.3470 (3)	0.43370 (19)	0.0219 (11)	
H47	0.8831	−0.3131	0.4658	0.026*	
C48	0.8939 (3)	−0.4531 (3)	0.42685 (18)	0.0166 (11)	
C49	0.8956 (4)	−0.5146 (3)	0.38202 (19)	0.0184 (11)	
H49	0.8963	−0.4876	0.3529	0.022*	
C50	0.8964 (3)	−0.6153 (3)	0.37934 (18)	0.0172 (11)	
H50	0.8962	−0.6552	0.3480	0.021*	
C51	0.8946 (4)	−0.6003 (3)	0.46190 (19)	0.0197 (11)	
H51	0.8944	−0.6294	0.4904	0.024*	
C52	0.8923 (4)	−0.4995 (3)	0.46770 (18)	0.0215 (11)	
H52	0.8895	−0.4616	0.4995	0.026*	
N42	0.8973 (3)	−0.6592 (3)	0.41862 (14)	0.0138 (8)	
N61	0.7475 (3)	0.1700 (3)	0.41465 (16)	0.0138 (9)	
C61	0.6980 (3)	0.1061 (3)	0.38163 (19)	0.0182 (11)	
H61	0.7340	0.0650	0.3577	0.022*	
C62	0.5987 (3)	0.0955 (4)	0.3798 (2)	0.0239 (12)	
H62	0.5681	0.0476	0.3556	0.029*	
C63	0.5432 (3)	0.1557 (4)	0.4137 (2)	0.0209 (11)	
C64	0.5928 (3)	0.2229 (4)	0.44899 (19)	0.0219 (11)	
H64	0.5583	0.2645	0.4733	0.026*	
C65	0.6929 (3)	0.2279 (3)	0.44797 (19)	0.0186 (11)	
H65	0.7257	0.2745	0.4720	0.022*	
C66	0.4368 (4)	0.1433 (4)	0.4108 (2)	0.0238 (12)	
H66	0.4098	0.0842	0.3930	0.029*	
C67	0.3763 (4)	0.2119 (4)	0.4321 (2)	0.0245 (12)	
H67	0.4030	0.2693	0.4517	0.029*	
C68	0.2702 (4)	0.2011 (4)	0.42626 (19)	0.0212 (12)	
C69	0.2193 (4)	0.1102 (4)	0.4201 (2)	0.0256 (12)	
H69	0.2538	0.0516	0.4196	0.031*	
C70	0.1206 (3)	0.1066 (3)	0.41500 (19)	0.0189 (11)	
H70	0.0887	0.0443	0.4101	0.023*	
C71	0.1145 (3)	0.2726 (3)	0.42231 (19)	0.0188 (11)	
H71	0.0787	0.3303	0.4225	0.023*	
C72	0.2137 (3)	0.2816 (4)	0.4281 (2)	0.0245 (12)	
H72	0.2437	0.3448	0.4334	0.029*	
N62	0.0656 (3)	0.1860 (3)	0.41647 (16)	0.0178 (9)	
S231	0.82516 (10)	0.17983 (10)	0.24772 (5)	0.0283 (3)	
C231	0.8693 (3)	0.1679 (3)	0.30103 (19)	0.0154 (10)	
N231	0.9001 (3)	0.1602 (3)	0.33914 (16)	0.0169 (9)	
S241	0.81912 (11)	0.16166 (11)	0.57629 (6)	0.0338 (4)	
C241	0.8690 (3)	0.1861 (3)	0.52692 (19)	0.0170 (11)	
N241	0.9043 (3)	0.2000 (3)	0.49035 (15)	0.0156 (9)	
Co3	0.88767 (4)	0.85152 (4)	0.08589 (2)	0.01137 (17)	
N81	1.0487 (3)	0.8598 (3)	0.09040 (15)	0.0175 (9)	
C81	1.0964 (3)	0.9469 (3)	0.09791 (19)	0.0186 (11)	
H81	1.0598	1.0045	0.0996	0.022*	
C82	1.1958 (4)	0.9568 (4)	0.1032 (2)	0.0250 (12)	
H82	1.2255	1.0201	0.1099	0.030*	
C83	1.2534 (3)	0.8730 (4)	0.0988 (2)	0.0199 (11)	
C84	1.2028 (3)	0.7837 (4)	0.09070 (19)	0.0186 (11)	
H84	1.2373	0.7246	0.0883	0.022*	
C85	1.1032 (3)	0.7804 (3)	0.08619 (18)	0.0182 (11)	
H85	1.0713	0.7181	0.0797	0.022*	
C86	1.3589 (3)	0.8843 (3)	0.1037 (2)	0.0205 (11)	
H86	1.3852	0.9416	0.1235	0.025*	
C87	1.4200 (4)	0.8179 (4)	0.0818 (2)	0.0240 (12)	
H87	1.3924	0.7597	0.0634	0.029*	
C88	1.5268 (3)	0.8274 (4)	0.0837 (2)	0.0188 (11)	
C89	1.5807 (3)	0.7675 (3)	0.0509 (2)	0.0216 (12)	
H89	1.5495	0.7195	0.0270	0.026*	
C90	1.6808 (3)	0.7766 (3)	0.05236 (19)	0.0203 (11)	
H90	1.7166	0.7328	0.0296	0.024*	
C91	1.6758 (3)	0.9026 (3)	0.11739 (19)	0.0194 (11)	
H91	1.7084	0.9490	0.1416	0.023*	
C92	1.5760 (3)	0.8987 (4)	0.11799 (19)	0.0206 (11)	
H92	1.5412	0.9430	0.1410	0.025*	
N82	1.7306 (3)	0.8451 (3)	0.08478 (15)	0.0148 (9)	
N101	0.8795 (3)	1.0095 (3)	0.08072 (14)	0.0132 (8)	
C101	0.8808 (4)	1.0387 (3)	0.0373 (2)	0.0231 (12)	
H101	0.8819	0.9904	0.0087	0.028*	
C102	0.8807 (4)	1.1355 (4)	0.0324 (2)	0.0284 (13)	
H102	0.8816	1.1523	0.0007	0.034*	
C103	0.8791 (3)	1.2093 (3)	0.07311 (19)	0.0184 (11)	
C104	0.8751 (4)	1.1785 (3)	0.11815 (19)	0.0196 (11)	
H104	0.8717	1.2251	0.1473	0.024*	
C105	0.8760 (3)	1.0795 (3)	0.12013 (18)	0.0168 (10)	
H105	0.8742	1.0603	0.1513	0.020*	
C106	0.8817 (4)	1.3122 (3)	0.0672 (2)	0.0254 (12)	
H106	0.8925	1.3250	0.0353	0.030*	
C107	0.8705 (4)	1.3891 (3)	0.1020 (2)	0.0224 (11)	
H107	0.8592	1.3766	0.1339	0.027*	
C108	0.8741 (3)	1.4911 (3)	0.09558 (19)	0.0181 (11)	
C109	0.8907 (4)	1.5237 (3)	0.0520 (2)	0.0234 (12)	
H109	0.8997	1.4776	0.0231	0.028*	
C110	0.8943 (4)	1.6224 (4)	0.05031 (19)	0.0216 (11)	
H110	0.9047	1.6420	0.0197	0.026*	
C111	0.8670 (4)	1.6611 (3)	0.13199 (19)	0.0213 (11)	
H111	0.8585	1.7086	0.1605	0.026*	
C112	0.8614 (4)	1.5637 (3)	0.1361 (2)	0.0242 (12)	
H112	0.8488	1.5458	0.1669	0.029*	
N102	0.8839 (3)	1.6922 (3)	0.08957 (15)	0.0144 (9)	
S251	0.83392 (13)	0.78391 (12)	−0.08676 (6)	0.0402 (4)	
C251	0.8672 (4)	0.8014 (3)	−0.0298 (2)	0.0184 (11)	
N251	0.8906 (3)	0.8150 (3)	0.01135 (17)	0.0193 (9)	
S261	0.81790 (12)	0.88533 (11)	0.25419 (5)	0.0355 (4)	
C261	0.8590 (3)	0.8888 (3)	0.20008 (19)	0.0159 (10)	
N261	0.8880 (3)	0.8892 (3)	0.16113 (16)	0.0166 (9)	
N121	0.4626 (4)	0.0743 (4)	0.2029 (2)	0.0469 (14)	
C121	0.5444 (5)	0.1086 (5)	0.2258 (2)	0.0438 (17)	
H121	0.6028	0.0772	0.2153	0.053*	
C122	0.5505 (4)	0.1869 (4)	0.2639 (2)	0.0317 (14)	
H122	0.6119	0.2095	0.2777	0.038*	
C123	0.4659 (4)	0.2328 (4)	0.2822 (2)	0.0292 (13)	
C124	0.3805 (5)	0.1963 (5)	0.2585 (3)	0.0473 (18)	
H124	0.3205	0.2251	0.2685	0.057*	
C125	0.3824 (5)	0.1188 (5)	0.2206 (3)	0.0532 (19)	
H125	0.3222	0.0950	0.2059	0.064*	
C126	0.4638 (4)	0.3162 (4)	0.3228 (2)	0.0287 (13)	
H126	0.4023	0.3432	0.3309	0.034*	
C127	0.5391 (4)	0.3571 (4)	0.3491 (2)	0.0296 (13)	
H127	0.6009	0.3305	0.3408	0.035*	
C128	0.5363 (4)	0.4401 (4)	0.3898 (2)	0.0310 (13)	
C129	0.4512 (4)	0.4870 (4)	0.4055 (2)	0.0291 (13)	
H129	0.3908	0.4656	0.3902	0.035*	
C130	0.4557 (4)	0.5650 (4)	0.4436 (2)	0.0378 (15)	
H130	0.3971	0.5972	0.4529	0.045*	
C131	0.6171 (5)	0.5531 (5)	0.4530 (3)	0.0484 (18)	
H131	0.6764	0.5754	0.4692	0.058*	
C132	0.6201 (4)	0.4757 (4)	0.4149 (2)	0.0395 (15)	
H132	0.6803	0.4465	0.4058	0.047*	
N122	0.5362 (4)	0.5986 (4)	0.4685 (2)	0.0426 (13)	
N141	0.5148 (5)	0.4053 (4)	0.0353 (2)	0.0557 (16)	
C141	0.4331 (5)	0.4408 (5)	0.0584 (3)	0.0479 (18)	
H141	0.3729	0.4098	0.0475	0.057*	
C142	0.4319 (5)	0.5180 (5)	0.0962 (2)	0.0415 (16)	
H142	0.3720	0.5399	0.1101	0.050*	
C143	0.5180 (4)	0.5642 (4)	0.1142 (2)	0.0324 (14)	
C144	0.6017 (5)	0.5289 (5)	0.0902 (3)	0.0474 (17)	
H144	0.6628	0.5588	0.1002	0.057*	
C145	0.5966 (5)	0.4511 (5)	0.0521 (3)	0.058 (2)	
H145	0.6554	0.4291	0.0369	0.070*	
C146	0.5247 (4)	0.6472 (4)	0.1545 (2)	0.0344 (14)	
H146	0.5876	0.6745	0.1629	0.041*	
C147	0.4519 (4)	0.6879 (4)	0.1802 (2)	0.0302 (13)	
H147	0.3889	0.6609	0.1718	0.036*	
C148	0.4596 (4)	0.7711 (4)	0.2206 (2)	0.0318 (14)	
C149	0.3772 (5)	0.8105 (4)	0.2447 (2)	0.0425 (16)	
H149	0.3149	0.7834	0.2347	0.051*	
C150	0.3855 (5)	0.8881 (5)	0.2827 (3)	0.058 (2)	
H150	0.3278	0.9122	0.2983	0.069*	
C151	0.5490 (5)	0.8926 (5)	0.2753 (3)	0.0464 (18)	
H151	0.6104	0.9210	0.2860	0.056*	
C152	0.5480 (5)	0.8170 (5)	0.2377 (3)	0.0417 (16)	
H152	0.6071	0.7949	0.2229	0.050*	
N142	0.4695 (4)	0.9316 (4)	0.2990 (2)	0.0459 (14)	
N161	0.5418 (4)	0.7374 (4)	0.3692 (2)	0.0466 (14)	
C161	0.4587 (5)	0.7713 (5)	0.3900 (3)	0.0468 (17)	
H161	0.3996	0.7400	0.3775	0.056*	
C162	0.4540 (4)	0.8481 (4)	0.4279 (2)	0.0367 (15)	
H162	0.3927	0.8681	0.4407	0.044*	
C163	0.5371 (4)	0.8967 (4)	0.4479 (2)	0.0286 (13)	
C164	0.6231 (5)	0.8625 (5)	0.4268 (3)	0.0476 (18)	
H164	0.6831	0.8933	0.4384	0.057*	
C165	0.6219 (5)	0.7843 (5)	0.3892 (3)	0.057 (2)	
H165	0.6825	0.7617	0.3764	0.068*	
C166	0.5390 (4)	0.9801 (4)	0.4884 (2)	0.0297 (13)	
H166	0.6006	1.0080	0.4991	0.036*	
N171	0.5214 (4)	0.2624 (4)	0.1331 (2)	0.0447 (14)	
C171	0.4422 (5)	0.2247 (5)	0.1081 (3)	0.0441 (17)	
H171	0.3821	0.2545	0.1169	0.053*	
C172	0.4410 (4)	0.1468 (4)	0.0709 (2)	0.0375 (15)	
H172	0.3812	0.1220	0.0560	0.045*	
C173	0.5280 (4)	0.1034 (4)	0.0548 (2)	0.0313 (14)	
C174	0.6106 (5)	0.1431 (5)	0.0799 (3)	0.0467 (18)	
H174	0.6721	0.1170	0.0707	0.056*	
C175	0.6042 (5)	0.2210 (5)	0.1183 (3)	0.0502 (19)	
H175	0.6623	0.2459	0.1350	0.060*	
C176	0.5358 (4)	0.0209 (4)	0.0139 (2)	0.0324 (13)	
H176	0.5986	−0.0043	0.0072	0.039*	

### Atomic displacement parameters (Å^2^)

**Table:**

	*U*^11^	*U*^22^	*U*^33^	*U*^12^	*U*^13^	*U*^23^
Co1	0.0127 (3)	0.0085 (3)	0.0109 (4)	−0.0014 (2)	0.0004 (2)	0.0019 (2)
N1	0.0120 (19)	0.0076 (18)	0.017 (2)	−0.0027 (14)	−0.0005 (16)	0.0045 (16)
C1	0.026 (3)	0.014 (2)	0.014 (3)	−0.001 (2)	−0.002 (2)	0.003 (2)
C2	0.032 (3)	0.015 (2)	0.017 (3)	0.002 (2)	−0.003 (2)	0.010 (2)
C3	0.026 (3)	0.008 (2)	0.014 (3)	−0.0035 (19)	−0.001 (2)	0.0066 (19)
C4	0.036 (3)	0.014 (2)	0.016 (3)	−0.001 (2)	0.000 (2)	0.003 (2)
C5	0.026 (3)	0.012 (2)	0.016 (3)	−0.0013 (19)	0.002 (2)	0.005 (2)
C6	0.042 (3)	0.010 (2)	0.017 (3)	0.000 (2)	−0.001 (2)	−0.002 (2)
C7	0.036 (3)	0.005 (2)	0.018 (3)	−0.002 (2)	−0.001 (2)	−0.003 (2)
C8	0.018 (2)	0.014 (2)	0.021 (3)	0.0040 (19)	0.006 (2)	0.004 (2)
C9	0.031 (3)	0.007 (2)	0.023 (3)	0.001 (2)	0.002 (2)	0.007 (2)
C10	0.028 (3)	0.011 (2)	0.014 (3)	−0.001 (2)	−0.002 (2)	0.0008 (19)
C11	0.024 (3)	0.011 (2)	0.017 (3)	−0.0014 (19)	0.001 (2)	0.003 (2)
C12	0.033 (3)	0.009 (2)	0.015 (3)	−0.004 (2)	0.000 (2)	−0.004 (2)
N2	0.0111 (19)	0.0134 (19)	0.016 (2)	0.0003 (15)	0.0010 (16)	0.0044 (17)
N21	0.014 (2)	0.014 (2)	0.016 (2)	−0.0030 (16)	−0.0018 (16)	0.0038 (17)
C21	0.018 (2)	0.010 (2)	0.019 (3)	0.0040 (19)	−0.002 (2)	−0.0019 (19)
C22	0.019 (3)	0.014 (3)	0.032 (3)	−0.005 (2)	0.001 (2)	0.005 (2)
C23	0.019 (3)	0.012 (2)	0.017 (3)	−0.0066 (19)	−0.005 (2)	0.006 (2)
C24	0.021 (3)	0.011 (2)	0.026 (3)	0.0025 (19)	0.001 (2)	−0.001 (2)
C25	0.019 (3)	0.016 (2)	0.011 (3)	−0.0051 (19)	0.002 (2)	−0.0025 (19)
C26	0.022 (3)	0.009 (2)	0.028 (3)	0.0033 (19)	0.003 (2)	−0.006 (2)
C27	0.018 (3)	0.016 (2)	0.025 (3)	−0.001 (2)	0.001 (2)	−0.001 (2)
C28	0.023 (3)	0.014 (2)	0.017 (3)	−0.009 (2)	0.001 (2)	0.003 (2)
C29	0.022 (3)	0.017 (2)	0.023 (3)	0.001 (2)	0.003 (2)	−0.007 (2)
C30	0.016 (3)	0.019 (3)	0.030 (3)	−0.002 (2)	0.001 (2)	0.001 (2)
C31	0.022 (3)	0.008 (2)	0.023 (3)	−0.0019 (19)	0.003 (2)	−0.001 (2)
C32	0.018 (3)	0.018 (3)	0.025 (3)	−0.007 (2)	−0.001 (2)	−0.002 (2)
N22	0.010 (2)	0.041 (3)	0.024 (3)	0.0029 (19)	0.0029 (18)	−0.005 (2)
S211	0.0493 (9)	0.0353 (8)	0.0207 (9)	0.0045 (7)	0.0094 (7)	0.0092 (6)
C211	0.020 (3)	0.011 (2)	0.024 (3)	0.0006 (19)	−0.004 (2)	0.002 (2)
N211	0.023 (2)	0.014 (2)	0.015 (3)	0.0004 (16)	−0.0011 (18)	−0.0004 (17)
S221	0.0513 (9)	0.0365 (8)	0.0162 (8)	0.0034 (7)	−0.0071 (7)	0.0077 (6)
C221	0.019 (2)	0.008 (2)	0.020 (3)	0.0010 (18)	0.003 (2)	0.0054 (19)
N221	0.021 (2)	0.0109 (19)	0.016 (3)	−0.0051 (16)	0.0033 (18)	0.0017 (16)
Co2	0.0119 (3)	0.0073 (3)	0.0106 (4)	−0.0013 (2)	0.0008 (2)	0.0007 (2)
N41	0.0119 (19)	0.0128 (19)	0.013 (2)	−0.0010 (15)	−0.0007 (16)	0.0025 (16)
C41	0.028 (3)	0.011 (2)	0.021 (3)	0.001 (2)	0.000 (2)	0.006 (2)
C42	0.043 (3)	0.005 (2)	0.016 (3)	0.003 (2)	0.000 (2)	−0.0029 (19)
C43	0.021 (3)	0.013 (2)	0.013 (3)	−0.0033 (19)	0.001 (2)	0.005 (2)
C44	0.032 (3)	0.007 (2)	0.019 (3)	−0.0035 (19)	0.000 (2)	0.005 (2)
C45	0.028 (3)	0.015 (2)	0.013 (3)	−0.002 (2)	0.001 (2)	0.000 (2)
C46	0.035 (3)	0.012 (2)	0.018 (3)	0.001 (2)	0.000 (2)	0.000 (2)
C47	0.040 (3)	0.010 (2)	0.014 (3)	−0.002 (2)	0.003 (2)	−0.002 (2)
C48	0.020 (3)	0.009 (2)	0.020 (3)	−0.0031 (18)	0.003 (2)	0.002 (2)
C49	0.028 (3)	0.010 (2)	0.018 (3)	−0.003 (2)	−0.001 (2)	0.006 (2)
C50	0.025 (3)	0.012 (2)	0.013 (3)	−0.0025 (19)	0.001 (2)	−0.0014 (19)
C51	0.029 (3)	0.011 (2)	0.019 (3)	−0.002 (2)	0.000 (2)	0.004 (2)
C52	0.042 (3)	0.010 (2)	0.011 (3)	−0.003 (2)	0.003 (2)	−0.0032 (19)
N42	0.015 (2)	0.0124 (19)	0.013 (2)	−0.0021 (15)	0.0025 (16)	−0.0018 (16)
N61	0.015 (2)	0.0058 (19)	0.020 (2)	0.0043 (15)	−0.0032 (17)	0.0006 (16)
C61	0.017 (3)	0.013 (2)	0.022 (3)	−0.0019 (19)	0.002 (2)	−0.002 (2)
C62	0.016 (3)	0.021 (3)	0.030 (3)	−0.005 (2)	0.002 (2)	−0.009 (2)
C63	0.015 (3)	0.025 (3)	0.024 (3)	−0.002 (2)	0.000 (2)	0.009 (2)
C64	0.016 (3)	0.027 (3)	0.021 (3)	0.008 (2)	0.004 (2)	−0.001 (2)
C65	0.018 (3)	0.012 (2)	0.023 (3)	−0.0051 (19)	−0.003 (2)	−0.002 (2)
C66	0.017 (3)	0.023 (3)	0.030 (3)	−0.002 (2)	−0.002 (2)	−0.001 (2)
C67	0.018 (3)	0.023 (3)	0.029 (3)	−0.005 (2)	−0.004 (2)	−0.004 (2)
C68	0.019 (3)	0.028 (3)	0.017 (3)	−0.001 (2)	0.004 (2)	0.005 (2)
C69	0.020 (3)	0.027 (3)	0.031 (4)	0.004 (2)	0.003 (2)	0.008 (2)
C70	0.017 (3)	0.017 (2)	0.022 (3)	−0.006 (2)	0.000 (2)	0.004 (2)
C71	0.017 (3)	0.018 (2)	0.021 (3)	−0.001 (2)	−0.001 (2)	0.003 (2)
C72	0.016 (3)	0.018 (3)	0.038 (4)	−0.003 (2)	−0.003 (2)	0.002 (2)
N62	0.013 (2)	0.023 (2)	0.017 (2)	0.0045 (17)	−0.0020 (17)	0.0029 (18)
S231	0.0384 (8)	0.0299 (7)	0.0178 (8)	0.0002 (6)	−0.0055 (6)	0.0077 (6)
C231	0.018 (2)	0.011 (2)	0.016 (3)	−0.0028 (18)	0.006 (2)	−0.0026 (19)
N231	0.022 (2)	0.011 (2)	0.017 (3)	−0.0034 (16)	0.0010 (19)	0.0025 (17)
S241	0.0427 (9)	0.0401 (9)	0.0215 (9)	−0.0036 (7)	0.0094 (6)	0.0130 (6)
C241	0.020 (3)	0.014 (2)	0.017 (3)	0.0011 (19)	−0.004 (2)	0.003 (2)
N241	0.024 (2)	0.0084 (19)	0.015 (3)	0.0010 (16)	−0.0018 (18)	0.0026 (16)
Co3	0.0125 (3)	0.0090 (3)	0.0128 (4)	−0.0003 (2)	0.0007 (2)	0.0023 (2)
N81	0.020 (2)	0.018 (2)	0.013 (2)	−0.0019 (17)	0.0024 (17)	0.0008 (17)
C81	0.018 (3)	0.013 (2)	0.025 (3)	0.0009 (19)	−0.003 (2)	0.002 (2)
C82	0.020 (3)	0.022 (3)	0.031 (3)	−0.007 (2)	0.002 (2)	0.000 (2)
C83	0.014 (3)	0.022 (3)	0.025 (3)	0.000 (2)	0.002 (2)	0.009 (2)
C84	0.022 (3)	0.015 (2)	0.019 (3)	0.004 (2)	0.003 (2)	0.004 (2)
C85	0.018 (3)	0.017 (3)	0.020 (3)	−0.002 (2)	−0.002 (2)	0.005 (2)
C86	0.015 (3)	0.017 (2)	0.028 (3)	−0.003 (2)	0.000 (2)	0.000 (2)
C87	0.017 (3)	0.023 (3)	0.032 (3)	−0.007 (2)	0.000 (2)	0.006 (2)
C88	0.018 (3)	0.017 (3)	0.020 (3)	−0.001 (2)	−0.001 (2)	0.000 (2)
C89	0.020 (3)	0.016 (2)	0.027 (3)	−0.008 (2)	−0.003 (2)	−0.002 (2)
C90	0.020 (3)	0.015 (2)	0.022 (3)	0.003 (2)	0.001 (2)	−0.008 (2)
C91	0.018 (3)	0.015 (2)	0.024 (3)	−0.003 (2)	−0.003 (2)	0.000 (2)
C92	0.018 (3)	0.020 (3)	0.021 (3)	0.002 (2)	0.005 (2)	−0.005 (2)
N82	0.025 (2)	0.0050 (19)	0.013 (2)	0.0008 (16)	−0.0027 (17)	−0.0011 (16)
N101	0.015 (2)	0.0127 (19)	0.013 (2)	−0.0002 (15)	0.0016 (16)	0.0073 (17)
C101	0.038 (3)	0.012 (2)	0.019 (3)	0.002 (2)	0.003 (2)	0.002 (2)
C102	0.055 (4)	0.014 (3)	0.019 (3)	0.002 (2)	0.009 (3)	0.008 (2)
C103	0.024 (3)	0.012 (2)	0.020 (3)	−0.0024 (19)	0.004 (2)	0.007 (2)
C104	0.026 (3)	0.013 (2)	0.019 (3)	0.000 (2)	0.001 (2)	0.000 (2)
C105	0.024 (3)	0.013 (2)	0.013 (3)	−0.0021 (19)	0.002 (2)	0.002 (2)
C106	0.044 (3)	0.012 (3)	0.020 (3)	0.002 (2)	0.004 (2)	0.004 (2)
C107	0.035 (3)	0.016 (3)	0.016 (3)	0.003 (2)	0.002 (2)	0.002 (2)
C108	0.021 (3)	0.011 (2)	0.022 (3)	0.0008 (19)	−0.005 (2)	0.001 (2)
C109	0.034 (3)	0.011 (2)	0.023 (3)	0.000 (2)	0.003 (2)	0.000 (2)
C110	0.028 (3)	0.021 (3)	0.017 (3)	0.004 (2)	0.004 (2)	0.005 (2)
C111	0.032 (3)	0.013 (2)	0.018 (3)	0.002 (2)	−0.002 (2)	0.002 (2)
C112	0.044 (3)	0.013 (2)	0.017 (3)	−0.003 (2)	0.000 (2)	0.007 (2)
N102	0.013 (2)	0.0136 (19)	0.017 (2)	0.0005 (15)	−0.0018 (16)	0.0040 (17)
S251	0.0628 (11)	0.0365 (9)	0.0205 (9)	0.0032 (7)	−0.0086 (7)	0.0028 (6)
C251	0.025 (3)	0.009 (2)	0.022 (3)	0.0019 (19)	0.005 (2)	0.004 (2)
N251	0.024 (2)	0.016 (2)	0.020 (3)	−0.0006 (17)	0.0048 (19)	0.0053 (18)
S261	0.0608 (10)	0.0308 (8)	0.0150 (8)	−0.0058 (7)	0.0115 (7)	0.0048 (6)
C261	0.021 (3)	0.010 (2)	0.016 (3)	−0.0002 (19)	−0.002 (2)	0.0026 (19)
N261	0.021 (2)	0.013 (2)	0.016 (3)	−0.0014 (16)	−0.0009 (18)	0.0032 (17)
N121	0.052 (4)	0.040 (3)	0.043 (4)	−0.006 (3)	0.006 (3)	−0.006 (3)
C121	0.048 (4)	0.042 (4)	0.037 (4)	0.007 (3)	0.015 (3)	−0.007 (3)
C122	0.038 (3)	0.023 (3)	0.032 (4)	0.001 (2)	0.006 (3)	−0.001 (3)
C123	0.039 (3)	0.022 (3)	0.025 (3)	−0.001 (2)	0.006 (3)	0.001 (2)
C124	0.031 (3)	0.050 (4)	0.056 (5)	0.001 (3)	0.002 (3)	−0.007 (3)
C125	0.043 (4)	0.046 (4)	0.061 (5)	−0.007 (3)	−0.004 (3)	−0.018 (4)
C126	0.031 (3)	0.026 (3)	0.028 (3)	0.001 (2)	0.008 (2)	0.001 (2)
C127	0.031 (3)	0.026 (3)	0.030 (4)	0.001 (2)	0.007 (3)	0.002 (2)
C128	0.030 (3)	0.026 (3)	0.034 (4)	−0.007 (2)	0.004 (3)	0.000 (3)
C129	0.034 (3)	0.028 (3)	0.025 (3)	0.000 (2)	−0.001 (2)	0.002 (2)
C130	0.038 (4)	0.029 (3)	0.042 (4)	0.006 (3)	0.002 (3)	−0.004 (3)
C131	0.040 (4)	0.045 (4)	0.052 (5)	−0.006 (3)	−0.005 (3)	−0.011 (3)
C132	0.035 (3)	0.038 (3)	0.043 (4)	0.008 (3)	0.000 (3)	0.001 (3)
N122	0.044 (3)	0.035 (3)	0.043 (4)	−0.002 (2)	0.000 (3)	−0.010 (2)
N141	0.066 (4)	0.046 (3)	0.047 (4)	−0.010 (3)	−0.013 (3)	−0.013 (3)
C141	0.055 (4)	0.038 (4)	0.045 (5)	−0.017 (3)	−0.016 (3)	−0.006 (3)
C142	0.045 (4)	0.037 (3)	0.038 (4)	−0.010 (3)	−0.013 (3)	−0.003 (3)
C143	0.043 (4)	0.022 (3)	0.030 (4)	−0.001 (2)	−0.009 (3)	0.002 (3)
C144	0.044 (4)	0.045 (4)	0.048 (5)	−0.004 (3)	−0.011 (3)	−0.007 (3)
C145	0.052 (4)	0.046 (4)	0.068 (6)	0.003 (3)	−0.010 (4)	−0.014 (4)
C146	0.040 (3)	0.028 (3)	0.033 (4)	−0.013 (3)	−0.011 (3)	0.002 (3)
C147	0.039 (3)	0.019 (3)	0.031 (4)	−0.007 (2)	−0.008 (3)	0.002 (2)
C148	0.039 (3)	0.027 (3)	0.029 (4)	−0.010 (2)	−0.006 (3)	0.007 (3)
C149	0.039 (4)	0.038 (4)	0.044 (4)	−0.011 (3)	−0.002 (3)	−0.011 (3)
C150	0.032 (4)	0.061 (5)	0.067 (6)	−0.011 (3)	0.001 (3)	−0.025 (4)
C151	0.035 (4)	0.041 (4)	0.056 (5)	−0.009 (3)	−0.004 (3)	−0.010 (3)
C152	0.034 (3)	0.049 (4)	0.039 (4)	−0.005 (3)	0.001 (3)	0.000 (3)
N142	0.043 (3)	0.042 (3)	0.046 (4)	−0.011 (2)	0.001 (3)	−0.008 (3)
N161	0.052 (4)	0.043 (3)	0.040 (4)	0.000 (3)	−0.001 (3)	−0.004 (3)
C161	0.046 (4)	0.039 (4)	0.050 (5)	−0.009 (3)	−0.010 (3)	−0.005 (3)
C162	0.035 (3)	0.033 (3)	0.037 (4)	0.000 (3)	−0.004 (3)	−0.006 (3)
C163	0.036 (3)	0.019 (3)	0.030 (4)	−0.001 (2)	−0.002 (3)	0.005 (2)
C164	0.033 (4)	0.045 (4)	0.058 (5)	−0.003 (3)	−0.003 (3)	−0.009 (3)
C165	0.039 (4)	0.052 (4)	0.073 (6)	0.006 (3)	0.007 (4)	−0.006 (4)
C166	0.032 (3)	0.023 (3)	0.035 (4)	−0.004 (2)	−0.007 (2)	0.005 (2)
N171	0.048 (3)	0.036 (3)	0.044 (4)	0.004 (2)	0.004 (3)	−0.010 (3)
C171	0.042 (4)	0.037 (4)	0.048 (5)	0.011 (3)	0.004 (3)	−0.009 (3)
C172	0.036 (3)	0.034 (3)	0.040 (4)	0.003 (3)	0.006 (3)	−0.001 (3)
C173	0.039 (3)	0.019 (3)	0.034 (4)	0.001 (2)	0.008 (3)	0.000 (2)
C174	0.033 (4)	0.049 (4)	0.050 (5)	0.005 (3)	0.005 (3)	−0.019 (3)
C175	0.034 (4)	0.036 (4)	0.070 (5)	−0.002 (3)	0.001 (3)	−0.019 (3)
C176	0.036 (3)	0.025 (3)	0.034 (4)	0.005 (2)	0.006 (3)	−0.001 (3)

### Geometric parameters (Å, º)

**Table:**

Co1—N211	2.063 (4)	N81—C81	1.348 (6)
Co1—N221	2.063 (4)	C81—C82	1.378 (7)
Co1—N22^i^	2.196 (4)	C81—H81	0.9500
Co1—N21	2.216 (4)	C82—C83	1.413 (7)
Co1—N2^ii^	2.216 (4)	C82—H82	0.9500
Co1—N1	2.245 (3)	C83—C84	1.393 (7)
N1—C1	1.333 (6)	C83—C86	1.463 (7)
N1—C5	1.350 (6)	C84—C85	1.378 (7)
C1—C2	1.394 (7)	C84—H84	0.9500
C1—H1	0.9500	C85—H85	0.9500
C2—C3	1.383 (7)	C86—C87	1.336 (7)
C2—H2	0.9500	C86—H86	0.9500
C3—C4	1.397 (7)	C87—C88	1.475 (7)
C3—C6	1.481 (6)	C87—H87	0.9500
C4—C5	1.380 (7)	C88—C89	1.364 (7)
C4—H4	0.9500	C88—C92	1.410 (7)
C5—H5	0.9500	C89—C90	1.383 (7)
C6—C7	1.299 (7)	C89—H89	0.9500
C6—H6	0.9500	C90—N82	1.363 (6)
C7—C8	1.481 (6)	C90—H90	0.9500
C7—H7	0.9500	C91—N82	1.351 (6)
C8—C12	1.386 (7)	C91—C92	1.377 (7)
C8—C9	1.394 (7)	C91—H91	0.9500
C9—C10	1.390 (6)	C92—H92	0.9500
C9—H9	0.9500	N82—Co3^i^	2.167 (4)
C10—N2	1.348 (6)	N101—C105	1.338 (6)
C10—H10	0.9500	N101—C101	1.339 (6)
C11—N2	1.340 (6)	C101—C102	1.376 (7)
C11—C12	1.388 (6)	C101—H101	0.9500
C11—H11	0.9500	C102—C103	1.392 (7)
C12—H12	0.9500	C102—H102	0.9500
N2—Co1^iii^	2.216 (4)	C103—C104	1.394 (7)
N21—C25	1.335 (6)	C103—C106	1.466 (7)
N21—C21	1.359 (6)	C104—C105	1.385 (7)
C21—C22	1.386 (7)	C104—H104	0.9500
C21—H21	0.9500	C105—H105	0.9500
C22—C23	1.391 (7)	C106—C107	1.325 (7)
C22—H22	0.9500	C106—H106	0.9500
C23—C24	1.415 (7)	C107—C108	1.458 (7)
C23—C26	1.469 (7)	C107—H107	0.9500
C24—C25	1.374 (7)	C108—C109	1.385 (7)
C24—H24	0.9500	C108—C112	1.393 (7)
C25—H25	0.9500	C109—C110	1.380 (7)
C26—C27	1.353 (7)	C109—H109	0.9500
C26—H26	0.9500	C110—N102	1.342 (6)
C27—C28	1.460 (7)	C110—H110	0.9500
C27—H27	0.9500	C111—N102	1.346 (6)
C28—C32	1.379 (7)	C111—C112	1.378 (7)
C28—C29	1.415 (7)	C111—H111	0.9500
C29—C30	1.383 (7)	C112—H112	0.9500
C29—H29	0.9500	N102—Co3^iii^	2.233 (4)
C30—N22	1.307 (7)	S251—C251	1.624 (6)
C30—H30	0.9500	C251—N251	1.170 (7)
C31—N22	1.360 (7)	S261—C261	1.623 (5)
C31—C32	1.375 (7)	C261—N261	1.160 (6)
C31—H31	0.9500	N121—C121	1.328 (9)
C32—H32	0.9500	N121—C125	1.336 (9)
N22—Co1^iv^	2.196 (4)	C121—C122	1.383 (9)
S211—C211	1.625 (6)	C121—H121	0.9500
C211—N211	1.179 (6)	C122—C123	1.400 (8)
S221—C221	1.619 (5)	C122—H122	0.9500
C221—N221	1.175 (6)	C123—C124	1.387 (9)
Co2—N241	2.075 (4)	C123—C126	1.475 (8)
Co2—N231	2.077 (4)	C124—C125	1.373 (9)
Co2—N61	2.167 (4)	C124—H124	0.9500
Co2—N62^i^	2.222 (4)	C125—H125	0.9500
Co2—N41	2.223 (4)	C126—C127	1.325 (8)
Co2—N42^iii^	2.231 (4)	C126—H126	0.9500
N41—C41	1.338 (6)	C127—C128	1.473 (8)
N41—C45	1.347 (6)	C127—H127	0.9500
C41—C42	1.378 (7)	C128—C132	1.383 (9)
C41—H41	0.9500	C128—C129	1.393 (8)
C42—C43	1.389 (7)	C129—C130	1.380 (8)
C42—H42	0.9500	C129—H129	0.9500
C43—C44	1.381 (7)	C130—N122	1.334 (8)
C43—C46	1.473 (6)	C130—H130	0.9500
C44—C45	1.382 (7)	C131—N122	1.335 (8)
C44—H44	0.9500	C131—C132	1.375 (9)
C45—H45	0.9500	C131—H131	0.9500
C46—C47	1.314 (7)	C132—H132	0.9500
C46—H46	0.9500	N141—C145	1.324 (9)
C47—C48	1.454 (6)	N141—C141	1.363 (9)
C47—H47	0.9500	C141—C142	1.367 (9)
C48—C49	1.387 (7)	C141—H141	0.9500
C48—C52	1.400 (7)	C142—C143	1.386 (8)
C49—C50	1.387 (6)	C142—H142	0.9500
C49—H49	0.9500	C143—C144	1.395 (9)
C50—N42	1.342 (6)	C143—C146	1.466 (8)
C50—H50	0.9500	C144—C145	1.378 (10)
C51—N42	1.337 (6)	C144—H144	0.9500
C51—C52	1.383 (7)	C145—H145	0.9500
C51—H51	0.9500	C146—C147	1.318 (9)
C52—H52	0.9500	C146—H146	0.9500
N42—Co2^ii^	2.231 (4)	C147—C148	1.470 (8)
N61—C61	1.334 (6)	C147—H147	0.9500
N61—C65	1.363 (6)	C148—C149	1.399 (9)
C61—C62	1.374 (7)	C148—C152	1.404 (8)
C61—H61	0.9500	C149—C150	1.377 (9)
C62—C63	1.397 (7)	C149—H149	0.9500
C62—H62	0.9500	C150—N142	1.334 (8)
C63—C64	1.395 (8)	C150—H150	0.9500
C63—C66	1.473 (7)	C151—C152	1.347 (9)
C64—C65	1.382 (7)	C151—N142	1.360 (8)
C64—H64	0.9500	C151—H151	0.9500
C65—H65	0.9500	C152—H152	0.9500
C66—C67	1.344 (7)	N161—C165	1.336 (9)
C66—H66	0.9500	N161—C161	1.345 (9)
C67—C68	1.474 (7)	C161—C162	1.370 (9)
C67—H67	0.9500	C161—H161	0.9500
C68—C72	1.370 (7)	C162—C163	1.380 (8)
C68—C69	1.414 (7)	C162—H162	0.9500
C69—C70	1.368 (7)	C163—C164	1.385 (9)
C69—H69	0.9500	C163—C166	1.472 (8)
C70—N62	1.347 (6)	C164—C165	1.374 (10)
C70—H70	0.9500	C164—H164	0.9500
C71—N62	1.351 (6)	C165—H165	0.9500
C71—C72	1.376 (7)	C166—C166^v^	1.340 (11)
C71—H71	0.9500	C166—H166	0.9500
C72—H72	0.9500	N171—C175	1.327 (8)
N62—Co2^iv^	2.222 (4)	N171—C171	1.338 (8)
S231—C231	1.635 (5)	C171—C172	1.362 (9)
C231—N231	1.162 (6)	C171—H171	0.9500
S241—C241	1.627 (5)	C172—C173	1.398 (8)
C241—N241	1.176 (6)	C172—H172	0.9500
Co3—N251	2.051 (5)	C173—C174	1.384 (9)
Co3—N261	2.071 (4)	C173—C176	1.476 (8)
Co3—N82^iv^	2.167 (4)	C174—C175	1.391 (9)
Co3—N81	2.223 (4)	C174—H174	0.9500
Co3—N101	2.228 (4)	C175—H175	0.9500
Co3—N102^ii^	2.233 (4)	C176—C176^vi^	1.311 (12)
N81—C85	1.338 (6)	C176—H176	0.9500
			
N211—Co1—N221	178.91 (15)	N251—Co3—N101	90.66 (15)
N211—Co1—N22^i^	89.41 (17)	N261—Co3—N101	89.11 (15)
N221—Co1—N22^i^	90.09 (17)	N82^iv^—Co3—N101	88.05 (14)
N211—Co1—N21	91.99 (15)	N81—Co3—N101	91.90 (14)
N221—Co1—N21	88.55 (15)	N251—Co3—N102^ii^	88.42 (15)
N22^i^—Co1—N21	177.48 (16)	N261—Co3—N102^ii^	91.90 (15)
N211—Co1—N2^ii^	90.75 (15)	N82^iv^—Co3—N102^ii^	87.70 (14)
N221—Co1—N2^ii^	90.18 (14)	N81—Co3—N102^ii^	92.39 (14)
N22^i^—Co1—N2^ii^	86.22 (16)	N101—Co3—N102^ii^	175.64 (14)
N21—Co1—N2^ii^	91.67 (14)	C85—N81—C81	116.5 (4)
N211—Co1—N1	90.22 (15)	C85—N81—Co3	122.7 (3)
N221—Co1—N1	88.82 (14)	C81—N81—Co3	120.8 (3)
N22^i^—Co1—N1	91.59 (16)	N81—C81—C82	123.4 (4)
N21—Co1—N1	90.49 (13)	N81—C81—H81	118.3
N2^ii^—Co1—N1	177.59 (14)	C82—C81—H81	118.3
C1—N1—C5	116.7 (4)	C81—C82—C83	120.1 (4)
C1—N1—Co1	122.3 (3)	C81—C82—H82	119.9
C5—N1—Co1	120.9 (3)	C83—C82—H82	119.9
N1—C1—C2	124.0 (5)	C84—C83—C82	115.6 (4)
N1—C1—H1	118.0	C84—C83—C86	124.6 (4)
C2—C1—H1	118.0	C82—C83—C86	119.7 (4)
C3—C2—C1	119.2 (4)	C85—C84—C83	120.4 (4)
C3—C2—H2	120.4	C85—C84—H84	119.8
C1—C2—H2	120.4	C83—C84—H84	119.8
C2—C3—C4	117.0 (4)	N81—C85—C84	123.9 (4)
C2—C3—C6	124.4 (4)	N81—C85—H85	118.1
C4—C3—C6	118.6 (4)	C84—C85—H85	118.1
C5—C4—C3	120.2 (5)	C87—C86—C83	123.3 (5)
C5—C4—H4	119.9	C87—C86—H86	118.3
C3—C4—H4	119.9	C83—C86—H86	118.3
N1—C5—C4	122.8 (4)	C86—C87—C88	125.6 (5)
N1—C5—H5	118.6	C86—C87—H87	117.2
C4—C5—H5	118.6	C88—C87—H87	117.2
C7—C6—C3	125.3 (5)	C89—C88—C92	118.1 (4)
C7—C6—H6	117.4	C89—C88—C87	119.9 (5)
C3—C6—H6	117.4	C92—C88—C87	122.0 (5)
C6—C7—C8	126.2 (5)	C88—C89—C90	120.1 (5)
C6—C7—H7	116.9	C88—C89—H89	120.0
C8—C7—H7	116.9	C90—C89—H89	120.0
C12—C8—C9	117.5 (4)	N82—C90—C89	123.4 (4)
C12—C8—C7	118.9 (5)	N82—C90—H90	118.3
C9—C8—C7	123.7 (4)	C89—C90—H90	118.3
C10—C9—C8	118.6 (5)	N82—C91—C92	124.5 (5)
C10—C9—H9	120.7	N82—C91—H91	117.8
C8—C9—H9	120.7	C92—C91—H91	117.8
N2—C10—C9	124.4 (5)	C91—C92—C88	118.4 (5)
N2—C10—H10	117.8	C91—C92—H92	120.8
C9—C10—H10	117.8	C88—C92—H92	120.8
N2—C11—C12	123.6 (4)	C91—N82—C90	115.5 (4)
N2—C11—H11	118.2	C91—N82—Co3^i^	123.4 (3)
C12—C11—H11	118.2	C90—N82—Co3^i^	120.9 (3)
C8—C12—C11	119.8 (5)	C105—N101—C101	116.7 (4)
C8—C12—H12	120.1	C105—N101—Co3	122.4 (3)
C11—C12—H12	120.1	C101—N101—Co3	120.8 (3)
C11—N2—C10	116.0 (4)	N101—C101—C102	123.0 (5)
C11—N2—Co1^iii^	120.9 (3)	N101—C101—H101	118.5
C10—N2—Co1^iii^	123.0 (3)	C102—C101—H101	118.5
C25—N21—C21	115.1 (4)	C101—C102—C103	120.9 (5)
C25—N21—Co1	122.4 (3)	C101—C102—H102	119.6
C21—N21—Co1	122.4 (3)	C103—C102—H102	119.6
N21—C21—C22	124.9 (4)	C102—C103—C104	116.0 (4)
N21—C21—H21	117.5	C102—C103—C106	120.2 (5)
C22—C21—H21	117.5	C104—C103—C106	123.8 (5)
C21—C22—C23	119.1 (4)	C105—C104—C103	119.6 (5)
C21—C22—H22	120.4	C105—C104—H104	120.2
C23—C22—H22	120.4	C103—C104—H104	120.2
C22—C23—C24	116.2 (4)	N101—C105—C104	123.8 (5)
C22—C23—C26	124.7 (4)	N101—C105—H105	118.1
C24—C23—C26	119.0 (4)	C104—C105—H105	118.1
C25—C24—C23	120.1 (4)	C107—C106—C103	126.7 (5)
C25—C24—H24	119.9	C107—C106—H106	116.7
C23—C24—H24	119.9	C103—C106—H106	116.7
N21—C25—C24	124.5 (4)	C106—C107—C108	126.0 (5)
N21—C25—H25	117.8	C106—C107—H107	117.0
C24—C25—H25	117.8	C108—C107—H107	117.0
C27—C26—C23	122.6 (4)	C109—C108—C112	115.7 (4)
C27—C26—H26	118.7	C109—C108—C107	125.4 (5)
C23—C26—H26	118.7	C112—C108—C107	118.9 (5)
C26—C27—C28	122.7 (5)	C110—C109—C108	120.6 (5)
C26—C27—H27	118.7	C110—C109—H109	119.7
C28—C27—H27	118.7	C108—C109—H109	119.7
C32—C28—C29	117.1 (5)	N102—C110—C109	123.5 (5)
C32—C28—C27	124.6 (5)	N102—C110—H110	118.2
C29—C28—C27	118.3 (4)	C109—C110—H110	118.2
C30—C29—C28	118.7 (5)	N102—C111—C112	123.0 (5)
C30—C29—H29	120.6	N102—C111—H111	118.5
C28—C29—H29	120.6	C112—C111—H111	118.5
N22—C30—C29	124.2 (5)	C111—C112—C108	120.9 (5)
N22—C30—H30	117.9	C111—C112—H112	119.6
C29—C30—H30	117.9	C108—C112—H112	119.6
N22—C31—C32	123.1 (5)	C110—N102—C111	116.3 (4)
N22—C31—H31	118.4	C110—N102—Co3^iii^	123.0 (3)
C32—C31—H31	118.4	C111—N102—Co3^iii^	120.7 (3)
C31—C32—C28	119.6 (5)	N251—C251—S251	179.2 (5)
C31—C32—H32	120.2	C251—N251—Co3	162.1 (4)
C28—C32—H32	120.2	N261—C261—S261	178.5 (4)
C30—N22—C31	117.1 (4)	C261—N261—Co3	155.5 (4)
C30—N22—Co1^iv^	121.9 (4)	C121—N121—C125	115.0 (6)
C31—N22—Co1^iv^	120.9 (3)	N121—C121—C122	124.6 (6)
N211—C211—S211	178.7 (5)	N121—C121—H121	117.7
C211—N211—Co1	161.2 (4)	C122—C121—H121	117.7
N221—C221—S221	179.0 (5)	C121—C122—C123	119.7 (6)
C221—N221—Co1	158.5 (4)	C121—C122—H122	120.1
N241—Co2—N231	178.11 (15)	C123—C122—H122	120.1
N241—Co2—N61	89.14 (16)	C124—C123—C122	115.5 (6)
N231—Co2—N61	89.11 (16)	C124—C123—C126	120.2 (5)
N241—Co2—N62^i^	89.70 (16)	C122—C123—C126	124.3 (5)
N231—Co2—N62^i^	92.07 (16)	C125—C124—C123	120.1 (6)
N61—Co2—N62^i^	178.36 (15)	C125—C124—H124	119.9
N241—Co2—N41	89.74 (14)	C123—C124—H124	119.9
N231—Co2—N41	90.91 (14)	N121—C125—C124	124.9 (6)
N61—Co2—N41	88.28 (14)	N121—C125—H125	117.5
N62^i^—Co2—N41	90.57 (14)	C124—C125—H125	117.5
N241—Co2—N42^iii^	89.30 (14)	C127—C126—C123	126.6 (5)
N231—Co2—N42^iii^	89.98 (14)	C127—C126—H126	116.7
N61—Co2—N42^iii^	89.52 (14)	C123—C126—H126	116.7
N62^i^—Co2—N42^iii^	91.62 (14)	C126—C127—C128	126.2 (5)
N41—Co2—N42^iii^	177.61 (14)	C126—C127—H127	116.9
C41—N41—C45	116.1 (4)	C128—C127—H127	116.9
C41—N41—Co2	120.1 (3)	C132—C128—C129	116.0 (6)
C45—N41—Co2	123.8 (3)	C132—C128—C127	120.8 (5)
N41—C41—C42	123.6 (5)	C129—C128—C127	123.2 (5)
N41—C41—H41	118.2	C130—C129—C128	119.2 (6)
C42—C41—H41	118.2	C130—C129—H129	120.4
C41—C42—C43	120.3 (5)	C128—C129—H129	120.4
C41—C42—H42	119.9	N122—C130—C129	125.1 (6)
C43—C42—H42	119.9	N122—C130—H130	117.5
C44—C43—C42	116.5 (4)	C129—C130—H130	117.5
C44—C43—C46	124.7 (4)	N122—C131—C132	124.1 (6)
C42—C43—C46	118.9 (4)	N122—C131—H131	118.0
C43—C44—C45	120.1 (5)	C132—C131—H131	118.0
C43—C44—H44	120.0	C131—C132—C128	120.6 (6)
C45—C44—H44	120.0	C131—C132—H132	119.7
N41—C45—C44	123.5 (5)	C128—C132—H132	119.7
N41—C45—H45	118.2	C130—N122—C131	115.0 (5)
C44—C45—H45	118.2	C145—N141—C141	115.2 (6)
C47—C46—C43	126.5 (5)	N141—C141—C142	124.5 (6)
C47—C46—H46	116.8	N141—C141—H141	117.8
C43—C46—H46	116.8	C142—C141—H141	117.8
C46—C47—C48	125.9 (5)	C141—C142—C143	120.0 (7)
C46—C47—H47	117.0	C141—C142—H142	120.0
C48—C47—H47	117.0	C143—C142—H142	120.0
C49—C48—C52	115.7 (4)	C142—C143—C144	115.7 (6)
C49—C48—C47	124.9 (4)	C142—C143—C146	124.3 (6)
C52—C48—C47	119.4 (4)	C144—C143—C146	120.0 (5)
C50—C49—C48	120.6 (4)	C145—C144—C143	120.8 (6)
C50—C49—H49	119.7	C145—C144—H144	119.6
C48—C49—H49	119.7	C143—C144—H144	119.6
N42—C50—C49	123.5 (5)	N141—C145—C144	123.9 (7)
N42—C50—H50	118.2	N141—C145—H145	118.0
C49—C50—H50	118.2	C144—C145—H145	118.0
N42—C51—C52	123.9 (5)	C147—C146—C143	126.4 (5)
N42—C51—H51	118.0	C147—C146—H146	116.8
C52—C51—H51	118.0	C143—C146—H146	116.8
C51—C52—C48	120.1 (5)	C146—C147—C148	125.8 (5)
C51—C52—H52	119.9	C146—C147—H147	117.1
C48—C52—H52	119.9	C148—C147—H147	117.1
C51—N42—C50	116.1 (4)	C149—C148—C152	115.2 (6)
C51—N42—Co2^ii^	120.1 (3)	C149—C148—C147	121.2 (5)
C50—N42—Co2^ii^	123.8 (3)	C152—C148—C147	123.5 (6)
C61—N61—C65	115.7 (4)	C150—C149—C148	120.7 (6)
C61—N61—Co2	121.5 (3)	C150—C149—H149	119.7
C65—N61—Co2	122.8 (3)	C148—C149—H149	119.7
N61—C61—C62	124.6 (5)	N142—C150—C149	124.1 (6)
N61—C61—H61	117.7	N142—C150—H150	118.0
C62—C61—H61	117.7	C149—C150—H150	118.0
C61—C62—C63	119.4 (5)	C152—C151—N142	125.5 (6)
C61—C62—H62	120.3	C152—C151—H151	117.2
C63—C62—H62	120.3	N142—C151—H151	117.2
C64—C63—C62	117.4 (4)	C151—C152—C148	119.9 (6)
C64—C63—C66	124.2 (5)	C151—C152—H152	120.1
C62—C63—C66	118.4 (5)	C148—C152—H152	120.1
C65—C64—C63	118.9 (5)	C150—N142—C151	114.6 (6)
C65—C64—H64	120.5	C165—N161—C161	114.5 (6)
C63—C64—H64	120.5	N161—C161—C162	124.2 (6)
N61—C65—C64	124.0 (5)	N161—C161—H161	117.9
N61—C65—H65	118.0	C162—C161—H161	117.9
C64—C65—H65	118.0	C161—C162—C163	120.9 (6)
C67—C66—C63	123.0 (5)	C161—C162—H162	119.6
C67—C66—H66	118.5	C163—C162—H162	119.6
C63—C66—H66	118.5	C162—C163—C164	115.5 (6)
C66—C67—C68	122.4 (5)	C162—C163—C166	124.6 (5)
C66—C67—H67	118.8	C164—C163—C166	119.9 (5)
C68—C67—H67	118.8	C165—C164—C163	120.1 (6)
C72—C68—C69	115.5 (5)	C165—C164—H164	119.9
C72—C68—C67	120.7 (5)	C163—C164—H164	119.9
C69—C68—C67	123.7 (5)	N161—C165—C164	124.9 (6)
C70—C69—C68	120.0 (5)	N161—C165—H165	117.6
C70—C69—H69	120.0	C164—C165—H165	117.6
C68—C69—H69	120.0	C166^v^—C166—C163	125.5 (7)
N62—C70—C69	124.1 (4)	C166^v^—C166—H166	117.3
N62—C70—H70	118.0	C163—C166—H166	117.3
C69—C70—H70	118.0	C175—N171—C171	115.5 (6)
N62—C71—C72	123.3 (4)	N171—C171—C172	125.0 (6)
N62—C71—H71	118.3	N171—C171—H171	117.5
C72—C71—H71	118.3	C172—C171—H171	117.5
C68—C72—C71	121.4 (5)	C171—C172—C173	119.9 (6)
C68—C72—H72	119.3	C171—C172—H172	120.1
C71—C72—H72	119.3	C173—C172—H172	120.1
C70—N62—C71	115.6 (4)	C174—C173—C172	115.5 (6)
C70—N62—Co2^iv^	123.3 (3)	C174—C173—C176	119.9 (5)
C71—N62—Co2^iv^	121.0 (3)	C172—C173—C176	124.5 (6)
N231—C231—S231	179.3 (5)	C173—C174—C175	120.5 (6)
C231—N231—Co2	156.4 (4)	C173—C174—H174	119.8
N241—C241—S241	177.3 (4)	C175—C174—H174	119.8
C241—N241—Co2	150.4 (4)	N171—C175—C174	123.6 (6)
N251—Co3—N261	178.71 (16)	N171—C175—H175	118.2
N251—Co3—N82^iv^	91.05 (16)	C174—C175—H175	118.2
N261—Co3—N82^iv^	90.21 (16)	C176^vi^—C176—C173	126.2 (7)
N251—Co3—N81	91.35 (16)	C176^vi^—C176—H176	116.9
N261—Co3—N81	87.39 (15)	C173—C176—H176	116.9
N82^iv^—Co3—N81	177.60 (16)		

Symmetry codes: (i) *x*+1, *y*, *z*; (ii) *x*, *y*−1, *z*; (iii) *x*, *y*+1, *z*; (iv) *x*−1, *y*, *z*; (v) −*x*+1, −*y*+2, −*z*+1; (vi) −*x*+1, −*y*, −*z*.
